# Operating Characteristics of a Tuberculosis Screening Tool for People Living with HIV in Out-Patient HIV Care and Treatment Services, Rwanda

**DOI:** 10.1371/journal.pone.0163462

**Published:** 2016-09-29

**Authors:** Kenneth Turinawe, Greet Vandebriel, David W. Lowrance, Francois Uwinkindi, Philippe Mutwa, Kimberly R. Boer, Grace Mutembayire, David Tugizimana, Sabin Nsanzimana, Eric Pevzner, Andrea A. Howard, Michel Gasana

**Affiliations:** 1 ICAP at Columbia University, Mailman School of Public Health, Kigali, Rwanda; 2 Division of Global HIV and TB, U.S. Centers for Disease Control and Prevention, Kigali, Rwanda; 3 Ministry of Health, Rwanda Biomedical Center/Institute of HIV Disease Prevention and Care, Kigali, Rwanda; 4 Global TB Branch, Division of Global HIV and TB, U.S. Centers for Disease Control and Prevention, Atlanta, Georgia, United States of America; 5 ICAP at Columbia University, Mailman School of Public Health, New York, New York, United States of America; McGill University, CANADA

## Abstract

**Background:**

The World Health Organization (WHO) 2010 guidelines for intensified tuberculosis (TB) case finding (ICF) among people living with HIV (PLHIV) includes a recommendation that PLHIV receive routine TB screening. Since 2005, the Rwandan Ministry of Health has been using a five-question screening tool. Our study objective was to assess the operating characteristics of the tool designed to identify PLHIV with presumptive TB as measured against a composite reference standard, including bacteriologically confirmed TB.

**Methods:**

In a cross-sectional study, the TB screening tool was routinely administered at enrolment in outpatient HIV care and treatment services at seven public health facilities. From March to September 2011, study enrollees were examined for TB disease irrespective of TB screening outcome. The examination consisted of a chest radiograph (CXR), three sputum smears (SS), sputum culture (SC) and polymerase chain reaction line-probe assay (Hain test). PLHIV were classified as having “laboratory-confirmed TB” with positive results on SS for acid-fast bacilli, SC on Lowenstein-Jensen medium, or a Hain test.

**Results:**

Overall, 1,767 patients were enrolled and screened of which; 1,017 (57.6%) were female, median age was 33 (IQR, 27–41), and median CD4^+^ cell count was 385 (IQR, 229–563) cells/mm^3^. Of the patients screened, 138 (7.8%) were diagnosed with TB of which; 125 (90.5%) were laboratory-confirmed pulmonary TB. Of 404 (22.9%) patients who screened positive and 1,363 (77.1%) who screened negative, 79 (19.5%) and 59 (4.3%), respectively, were diagnosed with TB. For laboratory-confirmed TB, the tool had a sensitivity of 54.4% (95% CI 45.3–63.3), specificity of 79.5% (95% CI 77.5–81.5), PPV of 16.8% and NPV of 95.8%.

**Conclusion:**

TB prevalence among PLHIV newly enrolling into HIV care and treatment was 65 times greater than the overall population prevalence. However, the performance of the tool was poorer than the predicted performance of the WHO recommended TB screening questions.

## Introduction

Tuberculosis (TB) alongside HIV remains a leading cause of morbidity and mortality worldwide. In 2014, there were an estimated 9.6 million new cases of TB and 1.5 million deaths from TB globally. Overall, 12% of TB cases were among people living with Human Immunodeficiency Virus (HIV). There has been important progress worldwide in implementing TB/HIV collaborative activities and the numbers of people in HIV care and treatment services screened for TB increased by 27% from 5.5 million to 7.0 million from 2013 to 2014. [1]

In Rwanda, the TB/HIV syndemic remains a public health priority. In 2014, the TB incidence rate in Rwanda was estimated at 63/100,000 population, with a case detection rate of 81%. Among people with TB tested for HIV, the prevalence was 25%.[1,2] Recent data from the national TB prevalence survey in Rwanda conducted in 2012 estimated a prevalence of bacteriologically confirmed TB of 119.3 per 100,000 population (95%CI 78.8–159.9). [3]

In 2005, the Rwandan Ministry of Health (MOH) responded to the growing TB/HIV syndemic by developing a national policy on TB/HIV collaborative programs,[4] establishing a TB/HIV working group (TB/HIV WG), and revised program guidelines. The national policy included guidelines for developing “one-stop” TB/HIV services including HIV counseling, testing, and ARV treatment of people with TB testing positive for HIV through the TB clinic. “One-stop” services also included systematic screening for TB disease among all people living with HIV (PLHIV).

The World Health Organization (WHO) 2010 guidelines for intensified tuberculosis (TB) case finding (ICF) among people living with HIV (PLHIV) includes a recommendation that PLHIV receive routine TB screening. Screening for TB disease among PLHIV is recommended to increase early detection and initiation of anti-TB treatment, and to prevent ongoing transmission in order to ultimately reduce TB-associated morbidity and mortality in the population. Regular TB screening is important at any stage of HIV-associated immunodeficiency, since HIV is a powerful risk factor for progression from latent TB infection (LTBI) to TB disease. TB screening remains important even after patients start antiretroviral therapy (ART) because PLHIV remain at increased risk of developing TB after initiating ART.[5] Similarly, TB screening among PLHIV is important to rule out TB disease before initiating Isoniazid Preventive Therapy (IPT) to prevent or treat LTBI and to subsequently avoid the risk of Isoniazid resistance due to inadvertent monotherapy.

In 2005, the Rwandan MOH began using a five-question symptom-based screening tool including cough ≥ two weeks, fever ≥ three weeks, weight-loss of ≥ three kg over four weeks, night sweats ≥ three weeks, and contact with someone known to have TB. In 2011, 93% of patients newly enrolled in HIV care and treatment at 403 facilities were screened for TB.[6] We aimed to assess the operating characteristics of the Rwandan TB screening tool designed to identify PLHIV with presumptive TB as measured against a composite reference standard, including bacteriologically confirmed TB.

## Methods

### Study Setting and Design

This validation study employed a cross-sectional design to assess the operating characteristics of a TB screening tool that was being implemented as a part of routine clinical practice. PLHIV attending an outpatient HIV care and treatment program at seven health facilities in Rwanda were screened for TB using the screening tool upon enrollment into HIV care. These seven health facilities (five district hospitals [DH] and two health centers [HC]) were selected because they were the largest HIV treatment sites based on monthly patient enrollment trends. These health facilities also represented outpatient HIV care and treatment sites that provided varying levels of TB diagnostic and treatment services in rural and urban health facilities in Rwanda.

The required sample size was determined to be 1,460 patients, assuming a TB prevalence of 5% among PLHIV newly enrolled into HIV care and treatment,[7] an alpha of 0.05% and power of 80% to detect a minimum difference of 2.5% in sensitivity between the screening questionnaire and reference standard. The study sample size was a priori increased by 20% to 1,752 to account for anticipated lost samples, breakage, and contamination.

### Study Population

Study participants included PLHIV age ≥ 21 years, and newly enrolled or transferred-in to HIV care and treatment services at the seven selected sites. For this study, a “newly enrolled” patient was defined as a patient who had undergone HIV testing with a positive test result, CD4^+^ cell count testing and completed clinical evaluation. Patients were defined as transferred-in if they were referred from an HIV care and treatment clinic other than one of the seven participating study sites. Pregnant women were included in the study. Patients were excluded if they were hospitalized, currently receiving treatment for TB, or awaiting results of an evaluation for TB.

### Study Procedures

#### Enrollment

Patients newly registered and transferring into HIV care and treatment at the seven sites were referred by clinic staff to the study coordinator based at each health facility. Patients were included if they met eligibility criteria and provided written informed consent.

#### Screening tool

All patients enrolled in the study were screened for TB, using the TB screening tool, by a trained nurse or doctor, as part of their routine care. The screening tool consisted of five TB symptom- and contact-based questions including cough (both any cough and cough for two weeks or more), night sweats for three weeks or more, weight loss of three kilograms or more in the previous four weeks, fever for three weeks or more and close contact with a known TB case. Any patient who replied ‘yes’ to at least one of the five questions was considered to have screened positive and presumed to have TB. “Any cough” was not included as an element in the national screening tool but was added for study purposes.

#### Diagnostic testing

All patients, regardless of positive or negative responses to the screening tool, provided three sputum samples (same day on the spot, next day early morning, and spot) which were then processed for smear microscopy for acid-fast bacilli (AFB) at the health facility laboratory. Patients who were unable to produce sputum spontaneously had sputum induced with nebulized hypertonic saline. Furthermore, using triple packaging, one of the three sputum samples, usually the same day spot sample, was sent to the National Reference Laboratory (NRL) located in Kigali for culture on solid media (Lowenstein Jensen) and further testing. Each patient also had a Posterior-Anterior (PA) and lateral chest radiograph taken. Chest radiographs were interpreted by two independent readers who were blinded to the results of the TB screening tool. In case of discordance, the two radiologists re-interpreted the radiograph together to reach a consensus.

#### Laboratory procedures

At the NRL, the samples were processed by use of *N*-Acetyl-L-cysteine and sodium hydroxide (NALC–NaOH), followed by centrifugation and then were re-suspended in 1.5 ml of phosphate. A portion of the sediment was stained and tested by AFB-smear microscopy (Auramine O staining). A second portion was cultured on solid Löwenstein–Jensen (LJ) media. Quality assurance of TB microscopy was ensured through the national system. Sputum slides from health centers were re-read by the laboratory technician at the district hospital. NRL staff supervised hospital-based testing and all discordant slides from the HC level.

Indirect drug-susceptibility testing (DST) using the proportion method on LJ solid medium was performed. Results of all smears, cultures and DST were delivered to the respective health facility-based service for use in patient care. If *M*. *tuberculosis* did not grow by eight weeks, the culture result was read as negative, and if the diagnosis of TB was still entertained, it was managed as culture-negative.

For all sputum collected for this study, additional diagnostic testing was planned to identify *M*. *tuberculosis*, using MPT64 antigen detection (Capilia TB®, Tauns Laboratories) or with the line-probe assay with the use of the Genotype MTBDR Plus assay (Hain Lifescience). However, not all study samples were tested using Capilia or Hain because of stock-outs.

#### Additional diagnostic testing

If a patient was clinically suspected of TB and required additional evaluation beyond that offered in the study, he or she proceeded with further TB assessment according to the national TB diagnostic guidelines.

#### Definition of composite reference standard for diagnostic *M tuberculosis*

Diagnosing TB can be complicated, and therefore an inclusive approach was taken. Based on a combination of laboratory tests and chest radiograph results, we defined cases of TB as either lab- confirmed or probable, and classified them based on site of disease as either pulmonary or extrapulmonary (Table 1).[8]

**Table 1:** TB Case Definitions.

For study purposes, the following case definitions were applied:	
**Laboratory-Confirmed Pulmonary TB**	One or more sputum smears positive for AFB or Sputum culture positive for *M*. *tuberculosis* by Lowenstein Jensen (growth of two or more colonies on solid media characterizing *M*. *tuberculosis*); or Hain positive; or Capillia with *M*. *tuberculosis* identified, if done.
**Probable Pulmonary TB**	Chest radiograph suggestive of TB after two series of three smears negative on microscopy, negative culture and negative HAIN, and no response to seven days of broad spectrum antibiotics. The CXR findings can be of classical pattern (upper lobe, bilateral infiltrates, cavitation, pulmonary fibrosis and shrinkage) or atypical pattern (interstitial infiltrates especially lower zones, intrathoracic lymphadenopathy, no cavitation and no abnormalities).
**Laboratory-Confirmed extra-pulmonary TB**	*M*. *tuberculosis* isolated from relevant site (e.g. lymph node, pleura, pericardium, peritoneum).
**Probable extra-pulmonary TB [9]**	Other diagnostic evidence of TB (caseation or granulomata or characteristic cerebrospinal fluid (CSF) changes). The CSF looks clear. The white cell count is less than 500 per mm^3^ with predominantly lymphocytes (or early in the course of infection, predominantly polymorphs). Protein level is high (0.6-2g/l) and the glucose low (less than 50% of glycaemia simultaneously measured).

#### Data analysis

Data analysis was conducted using STATA Version 12 (Copyright 1984–2007 StataCorp TX USA). Baseline characteristics are presented by means and medians as appropriate for parametric and non-parametric data; ANOVA/ Student’s T-test and Kruskil-Wallis / Mann Whitney U test were used to compare groups (TB confirmed, TB probable and TB negative, etc.). Comparisons of the statistical significance of differences between categorical data were done using the chi-square test.

Operating characteristics, including sensitivity, specificity, positive predictive value (PPV) and negative predictive value (NPV) with 95% CI and receiver operating curves (ROC), using a logistical model, were calculated for the TB screening tool (five screening questions together) and each question separately. Operating characteristics of the screening tool were determined based on the composite reference standard for confirmed TB cases and then confirmed and probable TB cases combined (which included those diagnosed by radiography).

Subgroup analysis on the added diagnostic value of the chest radiographs was completed using sensitivity and specificity compared to the composite reference standard for TB confirmed cases only (the probable cases were removed from this analysis as radiography was part of the case definition/composite reference standard).

Analyses were stratified by factors affecting operating characteristics (male versus female, urban versus rural areas, health center versus district/referral hospital, number of cases screened per site—above versus below the median, and CD4+ cell count <200 vs ≥200 cells/mm^3^).

#### Ethical issues

Study nurses obtained a signed consent form in Kinyarwanda from each study participant. There was no compensation for participants who screened positive for TB because all diagnostic follow-up was part of routine care. However, patients who screened negative were compensated for travel (3,000 RWF, equivalent of 5.5 USD) because of the additional health facility visits required to undergo diagnostic evaluation per the protocol.

This study was approved by the National AIDS Commission and Rwanda National Ethics Commission in Rwanda, and the Columbia University Institutional Review Board at Columbia University Medical Center. The study was determined to be non-research by CDC Atlanta based on the role of CDC investigators.

## Results

### Baseline Characteristics

**Fig 1:**
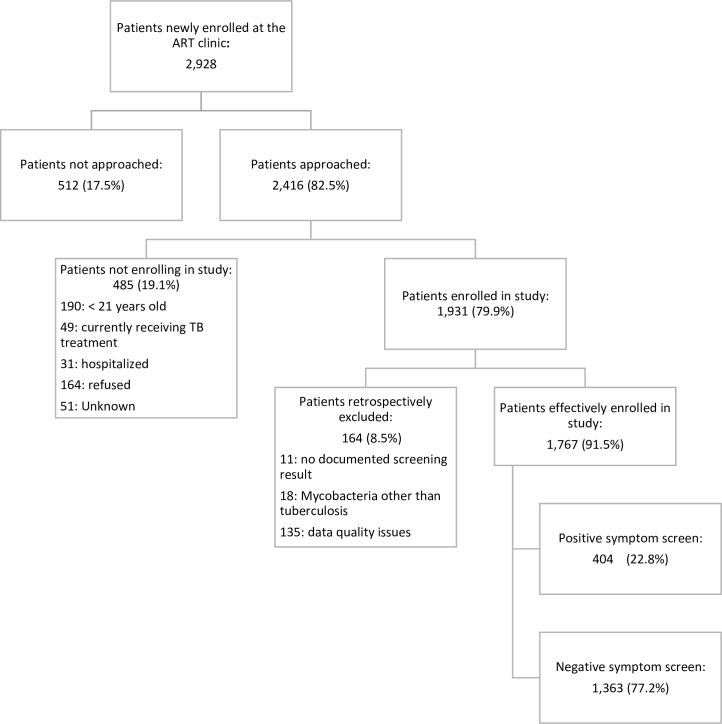
Flow Diagram of Patient Enrollment.

A total of 2,928 PLHIV sought HIV care and treatment at the seven study sites from March 1st, 2010 to September 16, 2011. Of these, 2,416 (82.5%) patients were referred to study staff. (Fig 1) Of those referred, 485 patients were excluded because they did not meet inclusion criteria: 190 (39.2%) were less than 21 years old, 49 (10.1%) were receiving anti-TB treatment, 31 (6.4%) were hospitalized, 164 (33.8%) refused participation, and for 51 (10.5%) the reason was not documented. In total, 65.9% (1,931/2,928) of the patients who sought HIV care were enrolled in the study, of whom 11 (0.6%) did not have documented screening results, 18 (0.9%) patients were excluded retrospectively because they were infected by mycobacteria other than TB (MOT) and 135 (7.0%) were removed from the analysis after enrollment in the study because of data quality issues and high levels of missing data.

Among the 1,767 participants, seven were transferred-in from other HIV care and treatment sites and were currently receiving ART; 937 (53.0%) were from Kigali, with the remainder from outside Kigali. (Table 2) The median age among the study population was 33 years (interquartile range [IQR] 27–41), and 1,017 (57.6%) of the patients were female. Based on clinical staging of HIV disease, 202 (11.7%) participants were classified as WHO stage III or IV. The median CD4+ cell count at enrollment was 385 cells/μL (IQR 229–563). Among 138 confirmed and probable TB cases, 62 (44.9%) were female, 57 (41.3%) had a CD4+ cell count <200 cells/μL and 23 (16.6%) were WHO stage III and IV. Kigali sites accounted for 74 (53.6%) of all TB cases identified in the study.

**Table 2:** Baseline Characteristics of Study Participants.

	Confirmed TB	Probable TB	No TB	Total
**N (%)**	125 (7.1)	13 (0.7)	1629 (92.2)	1767 (100)
Female gender, N (%)	59 (47.2)	3 (23.1)	955 (58.7)	1,017 (57.6)
Median age, y (IQR)	35 (28–41)	38 (26–44)	33 (27–41)	33 (27–41)
**CD4+ cell count, cells/mm**^**3**^				
Median (IQR)	304 (125–532)	151 (32–272)	395 (238–568)	385 (229–563)
<200, N (%)	48 (38.7)	9 (69.2)	324 (20.0)	381 (21.7)
> = 200, N (%)	76 (61.3)	4 (30.8)	1298 (80.0)	1378 (78.4)
**WHO stage**				
III & IV, N (%)	20 (16.4)	3 (25.0)	179 (11.3)	202 (11.7)
I & II, N (%)	102 (83.6)	9 (75.0)	1407 (88.7)	1518 (88.3)
Location				
Kigali, N (%)	67 (53.6)	7 (53.8)	863 (53.0)	937 (53.0)
Outside Kigali, N (%)	58 (46.4)	6 (46.2)	766 (47.0)	830 (47.0)
**Symptoms**				
Any cough, N (%)	64 (51.2)	9 (69.2)	337 (20.7)	410 (23.3)
Cough for 2 weeks or more, N (%)	53 (42.4)	7 (53.9)	143 (8.8)	203 (11.5)
Night sweats, N (%)	33 (26.4)	4 (30.8)	75 (4.6)	112 (6.4)
Fever, N (%)	29 (23.2)	5 (38.5)	47 (2.9)	81 (4.9)
Weight loss, N (%)	41 (32.8)	6 (46.2)	178 (11.0)	225 (12.7)
Contact with TB patient, N (%)	18 (14.6)	0 (0)	93 (5.7)	111 (6.3)
**Positive TB screen**^**a**^**, N (%)**	68 (54.4)	11 (84.6)	325 (20.0)	404 (22.9)

^a^Answered ‘yes’ to one of 5 questions on cough ≥ 2 weeks, weight loss of ≥ 3 kg in 4 weeks, night sweats for ≥ 3 weeks, fever for ≥ 3 weeks, contact with a known case of TB

### TB Screening Results

Twenty-three percent (n = 404) of the participants screened positive based on answering “yes” to at least one of the five questions on the screening tool. Of the 404 participants screening positive, 203 (50.2%) had cough for two weeks or more, 225 (55.7%) had experienced weight loss of three kg or more in the last four weeks, 81 (20.0%) had fever for three weeks or more, 112 (27.7%) presented with night sweats and 111 (27.5%) had been in contact with someone known to have TB disease. Among all participants screened, 410 (23.3%) were found to have any cough, regardless of the duration.

### TB Diagnostic Testing

**Fig 2:**
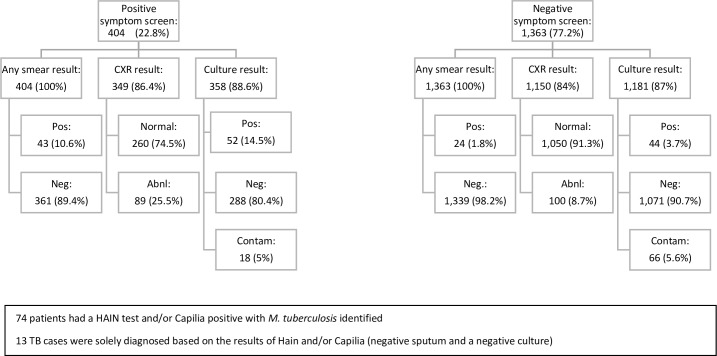
Diagnostic Testing.

All 404 of the participants who screened positive for TB had at least one smear microscopy result, of whom 43 (10.6%) were smear-positive cases. Of those who screened positive, 358 (88.6%) had a sputum culture done at the NRL of which 52 (14.5%) were positive for *M*. *tuberculosis*. Anterior-posterior and lateral chest radiographs were taken for 349 (86.4%) participants screening positive, of which 89 (25.5%) were classified as abnormal. (Fig 2).

Of the 1,363 participants who answered “no” to all five screening questions, 100% had at least one sputum smear, of whom 24 (1.8%) were sputum smear-positive. Eighty-seven percent (1,181) had cultures performed with 44 (3.7%) positive for *M*. *tuberculosis*. Of the 1,150 (84.4%) patients who underwent a CXR, 100 (8.7%) were interpreted as abnormal.

Overall, 138 (7.8%) of 1,767 participants enrolled were diagnosed with TB, of which 125 (90.6%) were laboratory-confirmed pulmonary TB cases. Of the lab-confirmed cases, 74 (59%) participants had a positive Hain and/or Capilia test, of which 13 (10.4%) were diagnosed only with Hain/Capilia and the remaining cases were diagnosed with a combination of sputum smear examination and or culture. Forty (29%) participants had negative sputum smear results but a positive culture for *M*. *tuberculosis*. Of the 13 participants defined as probable cases of TB, 10 (7.2%) were pulmonary and three were extra-pulmonary (one with miliary TB and two with pleural TB). These participants with probable cases of TB had negative sputum smears and culture (Hain test was not done). All 13 participants diagnosed with probable TB cases presented with clinical symptoms and a chest radiograph determined to be suggestive of TB.

### Operating Characteristics of the Screening Tool

Of the 404 participants who screened positive, 68 (16.8%) had laboratory-confirmed TB. Of the 1,363 participants who screened negative, 57 (4.2%) had laboratory-confirmed TB, including 27 with a positive culture but negative smears. The sensitivity of the national screening tool for laboratory confirmed TB cases was 54.4% (95% CI: 45.3%–63.3%) and increased to 67.8% (95% CI: 57.9%–76.3%) by replacing “cough for two weeks” with “any cough” and adding a CXR to the screening tool. The specificity of the tool was 79.5% (95% CI: 77.5%–81.5%) and decreased to 65.3% (95% CI: 62.7%–67.8%) after replacing the “cough for two weeks” with “any cough” and adding a CXR (Table 3).

**Table 3:** Operating Characteristics of the National Screening Tool, by Screening Tool Element.

	Sensitivity	Specificity	Positive Predictive Value	Negative Predictive Value
**125 confirmed TB cases**				
National screening tool	54.4 (45.3–63.3)	79.5 (77.5–81.5)	16.8 (13.3–20.8)	95.8 (94.6–96.8)
National screening tool replacing cough ≥ 2 weeks with any cough	58.4 (49.2–67.1)	70.8 (68.5–73.0)	13.2 (10.5–16.3)	95.7 (94.4–96.8)
National screening tool and chest X-ray	63.2 (54.91–71.6)	74.2 (72.0–76.3)	15.7 (12.6–19.2)	96.4 (95.2–97.3)
National screening tool replacing cough ≥ 2 weeks with any cough and chest X-ray	67.8 (57.9–76.3)	65.3 (62.7–67.8)	13.3 (10.6–16.4)	96.2 (94.8–97.4)
Cough ≥ 2 weeks	42.4 (33.6–51.6)	90.8 (89.3–92.2)	26.1 (20.2–32.7)	95.4 (94.3–96.4)
Any cough	51.2 (42.1–60.2)	78.9 (76.9–80.8)	15.6 (12.2–19.5)	95.5 (94.2–96.5)
Fever ≥ 3 weeks	23.2 (16.1–31.6)	96.8 (95.9–97.6)	35.8 (25.4–47.2)	94.3 (93.1–95.4)
Night sweats ≥ 3 weeks	26.8 (19.2–35.6)	95.2 (94.0–96.2)	29.5 (21.2–38.8)	94.5 (93.3–95.6)
Contact with TB case	14.6 (8.9–22.1)	94.3 (93.1–95.4)	16.2 (9.9–24.4)	93.6 (92.3–94.8)
Weight loss ≥ 3 kg in 4 weeks	32.8 (24.7–41.8)	88.8 (87.1–90.3)	18.2 (13.4–23.9)	94.5 (93.3–95.6)
**138 confirmed and probable TB cases**				
National screening tool^a^	57.2 (48.5–65.6)	80.0 (78.0–82.0)	19.6 (15.8–23.8)	95.7 (94.5–96.7)
National screening tool replacing cough ≥ 2 weeks with any cough	60.9 (52.2–69.1)	71.3 (69.0–73.5)	15.2 (12.3–18.5)	95.6 (94.2–96.6)
Cough ≥ 2 weeks	43.5 (35.1–52.2)	91.3 (89.7–92.5)	29.6 (23.4–36.3)	95.0 (93.8–96.0)
Any cough	52.9 (44.2–61.4)	79.3 (77.2–81.2)	17.8 (14.2–21.9)	95.2 (93.9–96.3)
Fever ≥ 3 weeks	24.6 (17.7–32.7)	97.1 (96.2–97.9)	42.0 (31.1–53.5)	93.9 (92.6–95.0)
Night sweats ≥ 3 weeks	27.2 (19.9–35.3)	95.4 (94.2–96.4)	33.0 (24.2–42.6)	94.0 (92.7–95.1)
Contact with TB case	13.2 (8.0–20.0)	94.3 (93.0–95.4)	16.2 (9.9–24.4)	92.8 (91.5–94.0)
Weight loss ≥ 3 kg in 4 weeks	34.1 (26.2–42.6)	89.1 (87.4–90.5)	20.9 (15.8–26.8)	94.1 (92.8–95.2)

^a^Questions on cough ≥ 2 weeks, weight loss of ≥ 3 kg in 4 weeks, night sweats for ≥ 3 weeks, fever for ≥ 3 weeks, contact with a known case of TB

Of the 13 participants diagnosed with probable TB, 11 (84.6%) screened positive and two were missed using the screening tool. When combining the confirmed and probable TB cases, the sensitivity of the national screening tool was 57.2% (95% CI: 48.5%–65.6%) and the specificity was 80.0% (95% CI: 78.0%–82.0%).

Significant differences in the performance of the tool among participant subgroups were observed (Table 4). When stratifying by gender, the tool had sensitivity of 43.5% in women compared to 68.4% in men and specificity of 82.6% and 76.5%, respectively. For patients with a CD4 cell count <200 cells/mm^3^, the tool has sensitivity and specificity of 71.9% (95% CI: 58.5%–83.0%) and 68.8% (95% CI 63.5%–73.8%) and for patients with CD4 cell count ≥200 46.3% (95% CI: 35.0%–57.8%) and 82.8% (95% CI: 80.6%–84.8%) respectively. Similarly, when used for patients at WHO stage III or IV the tool had a specificity of 62.6% (95% CI: 55.0%–69.7%) compared to 82.1% (95% CI: 80.0%–84.1%) for patients with WHO stage I and II. The highest sensitivity of 78.3% (56.3–92.5) was found among participants with WHO clinical stage III & IV and those with a CD4+ count <200 cells/mm^3^ 71.9% (95% CI: 58.5–83.0).

**Table 4:** Operating Characteristics of the National TB Screening Tool Stratified by Participant and Facility Characteristics for Confirmed and Probable TB Cases Combined.

	Sensitivity % (95% CI)	Specificity % (95% CI)	Positive Predictive Value % (95% CI)	Negative Predictive Value % (95% CI)
**Location:**				
Kigali	63.5 (51.5–74.4)	85.3 (82.7–87.6)	27.0 (20.6–34.3)	96.5 (94.9–97.7)
Outside of Kigali	50.0 (37.2–62.8)	74.2 (70.9–77.2)	13.9 (9.72–19.1)	94.7 (92.6–96.3)
**Health facility**				
Hospitals	58.3 (47.8–68.3)	77.5 (74.9–80.0)	19.2 (14.8–24.2)	95.3 (93.7–96.6)
Health Centers	51.3 (34.8–67.6)	87.3 (83.3–90.5)	30.8 (19.9–43.4)	94.2 (91.1–96.5)
**Gender**				
Male	68.4 (56.7–78.6)	76.5 (73.1–79.6)	24.8 (19.1–31.2)	95.5 (93.5–97.1)
Female	43.5 (31.0–56.7)	82.6 (80.1–85.0)	14.0 (9.43–19.7)	95.8 (94.1–97.0)
**CD4+ count**				
< 200 cells/mm^3^	71.9 (58.5–83.0)	68.8 (63.5–73.8)	28.9 (21.6–37.1)	93.3 (89.4–96.1)
≥ 200 cells/mm^3^	46.3 (35.0–57.8)	82.8 (80.6–84.8)	14.2 (10.2–19.1)	96.2 (94.8–97.2)
**WHO stage**				
III & IV	78.3 (56.3–92.5)	62.6 (55.0–69.7)	21.2 (13.1–31.4)	95.7 (90.3–98.6)
I & II	54.1 (44.3–63.6)	82.1 (80.0–84.1)	19.3 (15.1–24.1)	95.8 (94.5–96.8)

When analyzing the performance of each of the screening questions for the confirmed cases only or when the confirmed and probable TB cases were combined, no single indicator achieved a sensitivity of greater than 55% (Table 3). History of cough, regardless of duration, achieved a sensitivity of 51.2%, with a specificity of 78.9% for the confirmed cases and a sensitivity of 52.9%, with a specificity of 79.3% for confirmed and probable cases combined. Cough ≥ two weeks’ duration had a sensitivity of 42.4% with a specificity of 90.8% compared to a sensitivity of 43.5% with a specificity of 91.3% for confirmed only or confirmed and probable cases combined, respectively.

**Fig 3:**
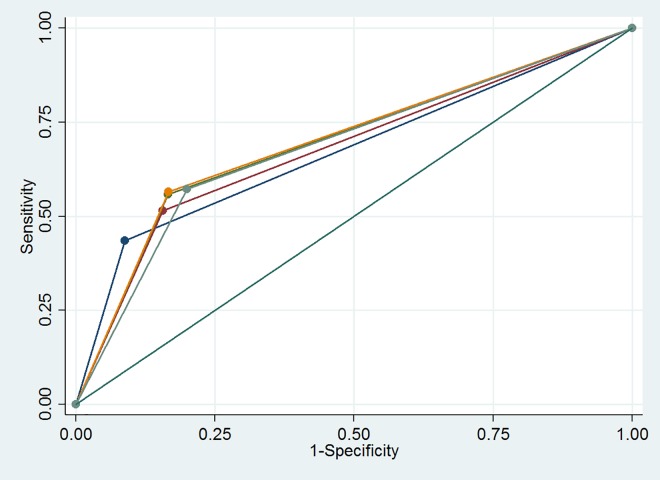
Receiver Operating Curves (ROC) with Area under the Curve (AUC) for each component of the screening tool with any cough and chest x-ray for confirmed cases only (n = 125). Dark blue: Any cough, red: Any Cough + weight loss, green: Any Cough + weight loss + night sweats ≥3 weeks, orange: Any Cough + weight loss + night sweats ≥3 weeks + fever ≥3 weeks, light bleu: Any Cough + weight loss + night sweats ≥3 weeks +s fever ≥3 weeks + known TB contact, F: any cough + weight loss + night sweats ≥3 weeks + fever ≥3 weeks + TB contact + x-ray.

**Fig 4:**
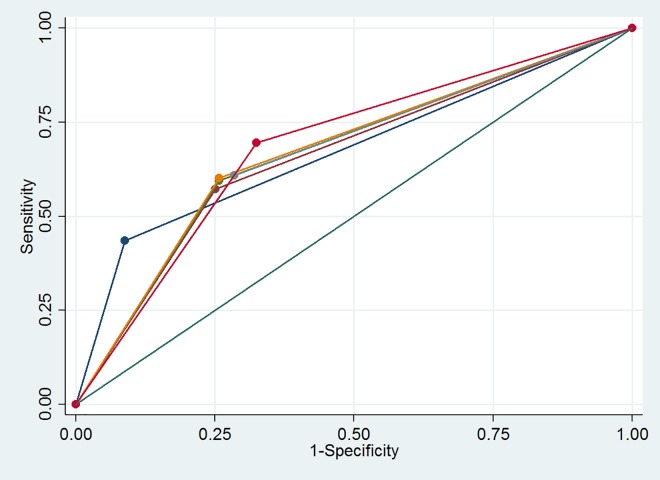
Receiver Operating Curves (ROC) with Area under the Curve (AUC) for each component of the screening tool for confirmed and probable cases (n = 138). Dark blue: Cough ≥2 weeks, brown: Cough ≥2 weeks + weight loss, green: Cough ≥2 weeks + weight loss + night sweats ≥3 weeks, orange: Cough ≥2 weeks + weight loss + night sweats ≥3 weeks + fever ≥3 weeks, light blue: Cough ≥2 weeks + weight loss + night sweats ≥3 weeks + fever ≥3 weeks + known TB contact, red: Cough ≥2 weeks + weight loss + night sweats ≥3 weeks + fever ≥3 weeks + TB contact.

In Fig 3 receiver operating curves (ROC) are presented for confirmed cases to demonstrate the added value of each additional symptom. “Any cough” was the strongest predictor, with an area under the curve (AUC) of 0.65 (95% CI: 0.60–0.69); by adding weight loss, the AUC didn’t change, and adding night sweats the AUC was 0.66 (95% CI: 0.61–0.70). The other screening questions added little value to the operating characteristics. Fig 4 shows the receiver operating curves for the confirmed and probable cases combined. Cough for two weeks or more was the strongest predictor, with an AUC of 0.67 (95% CI: 0.63–0.72); by adding weight loss, the AUC increased marginally to 0.68 (95% CI: 0.63–0.72), while adding night sweats increased the AUC to 0.70 (95% CI: 0.65–0.74). By replacing cough for more than two weeks with “any cough” the overall AUC did not change. Other screening questions added little value to the operating characteristics.

## Discussion

This study aimed to understand the performance of the Rwandan national TB screening tool among PLHIV enrolling in out-patient HIV care and treatment settings. During the study enrollment period in 2011, WHO published updated guidelines for intensified TB case finding among PLHIV, which recommend a four-element symptom-based screening tool consisting of any current cough, fever, night sweats, or weight loss.[10] The 54.4% sensitivity of the Rwandan national screening tool for confirmed TB cases was much lower than that of the WHO recommended tool (78.9%).[11] Although the NPV of 95.8% was only slightly lower than that of the WHO-recommended tool, the overall performance of the Rwandan tool was poor in comparison with international normative guidance. These findings support revision of the national TB screening tool to adapt it to the WHO recommended tool and take out the temporality of symptoms which was undertaken in 2012.

TB screening tools are developed to have a high sensitivity and negative predictive value to identify people suspected of having TB who require additional diagnostic testing and avoid false negatives.[10] Screening tools can allow for many false positives as confirmatory testing is typically part of the clinical algorithm. In this evaluation, the national screening tool showed a low sensitivity (54.4%), and specificity (79.5%). Fifty-nine participants who screened negative were later diagnosed with confirmed or probable TB. However, the tool had a high NPV (95.8% for confirmed and probable TB), indicating that absence of all of the components of the tool can select a subset of PLHIV who have low probability of having TB disease and in whom isoniazid preventive therapy can be initiated once TB is excluded.

The overall prevalence of TB in PLHIV newly enrolled in HIV care and treatment in this study was higher (7.8%) than expected based on routine monitoring and evaluation data from Rwanda. Routine programmatic data from Rwandan HIV care and treatment clinics since 2006 suggest a prevalence of TB disease of about 2% among PLHIV.[12] The higher observed prevalence in this study is probably due to a combination of factors, including the additional diagnostic testing performed regardless of screening results, as well as likely under-reporting and incomplete diagnostic workup for people suspected of having TB under routine programmatic circumstances. However, the prevalence was comparable to what has been reported for PLHIV in similar settings. Authors of a meta-analysis which informed the development of the WHO-recommended screening tool for TB in PLHIV reported that based on 12 studies with 8,148 patients in resource constrained settings the overall prevalence of TB disease among PLHIV was 5.8% (557/9,626), ranging from 0.4% to 25.7%.[11] The higher prevalence of TB disease among PLHIV in this study indicates the need for better screening algorithms, use of more sensitive and rapid TB diagnostics such as GeneXpert, along with robust linkages to TB services.[13]

The proportion of probable pulmonary TB and extra-pulmonary cases was lower than expected in a population newly enrolled in HIV care.[14,15] It has been well documented that as immunosuppression progresses, commensurate with CD4^+^ cell count decline, the proportion of smear negative pulmonary TB and extra-pulmonary TB cases increases. In this sample, patients initially had relatively high median CD4^+^ cell counts (385 cells/mm^3^). For participants with confirmed TB, the proportion with CD4^+^ cell count ≥200 cells/mm^3^ was 61.3%, while in smear negative and extra-pulmonary (probable) TB the proportion was 30.8%. The low proportion of smear-negative pulmonary tuberculosis and extra-pulmonary tuberculosis cases observed in this study may be a function of participants registering for HIV care when CD4^+^ cell counts were relatively high and that nearly 90% of the study population had a WHO clinical staging of I or II. Also of note, the HIV program in Rwanda has matured as evidenced by the national trend of increasing median baseline CD4+ cell counts in patients enrolling in HIV care.[16]

Of the different components of the screening tool, cough for two weeks or more was the strongest predictor of TB (AUC 0.67). This is mainly due to the fact that most of the cases were pulmonary TB (98%). Cough for two weeks or more as a single symptom was poorly sensitive consistent with earlier studies conducted in Ethiopia, Zimbabwe and South East Asia.[17,18,19] Using the symptom “any cough” instead of “cough for two weeks or more” improved the sensitivity of the tool in all settings but remained lower than expected. In some earlier studies, using a combination of symptoms and removing the temporality of all the symptoms (e.g. remove two weeks and replace with any time period) increased the overall sensitivity of the screening tool to more than 90%.[19,20] In our study, we observed a marginal difference in sensitivity by adding the other screening questions; however, this may be due to the fact that we only removed temporality for the cough component, while all other symptom components required three or four weeks duration to be recorded as positive.

Changing cough of two weeks or more to any cough and adding the results of the chest X-ray improved sensitivity of the tool from 54.4% to 67.8%. However, the specificity dropped to 65.3% and PPV to 13.3% while the negative predictive value remained high at 96.2%. These results are similar to other published reports, although there were variations in screening tool components and populations screened within the studies.[11] The added value of the chest x-ray in our study was only determined in confirmed cases (because a suggestive x-ray was part of the definition of probable TB case, which would artificially inflate the sensitivity). In this study we observed an 8.8% increase in sensitivity from 54.4% to 63.2% in confirmed cases of TB after adding chest X-ray findings to the national screening tool. This increase is consistent with the 11.7% increase noted from a meta-analysis including 12 studies and over 8,000 participants where the authors analyzed the yield of adding chest radiography to a four symptom screen (i.e., current cough, fever night sweats and weight loss) for screening for TB disease.[11]

The NPV of the national tool was high which made the tool appropriate for excluding active TB prior to initiation of IPT. This was also observed in other studies in Ethiopia, Zimbabwe and South East Asia.[16,18,19] However, replacing “cough for two weeks or more” by “any cough” and adding a chest X-ray resulted in a marginal increase in the NPV of 0.4% (from 95.8% to 96.2%).

This study had several limitations. There was initial training and periodic supervision of study nurses however variability in use of the screening tool within and across sites was not systematically evaluated. The screening tool may not have been applied consistently and uniformly across sites. Also, although temporality of cough was removed for the analysis of the additional yield for screening, this was not recorded for other screening components (fever, night sweats and weight loss) as recommended by the WHO guidelines for intensified TB case finding that were issued after our evaluation protocol was implemented.^7^ Unfortunately because of stock-outs at the NRL, study staff were unable to perform Hain and/or Capilia on all samples. In addition, cultures were done on solid media in accordance with national guidelines which is known to be less sensitive than culture performed using liquid media.[20]

Overall, the Rwandan TB screening tool that included temporal parameters performed poorly compared with the predicted performance of the WHO-recommended screening questions. This finding reinforces the current international recommendations—and current, revised Rwandan national guidelines—to assess for the presence and not the duration of symptoms when screening for TB among PLHIV. Also, our study demonstrates the importance of routine TB screening among PLHIV. Using a screening tool with relatively poor operating characteristics we were still able to document a TB prevalence among PLHIV that was 65 times greater than what is observed in the general population in Rwanda.
